# Diagnostic value of thyroid imaging reporting and data system combined with BRAF^V600E^ mutation analysis in Bethesda categories III–V thyroid nodules

**DOI:** 10.1038/s41598-022-09822-5

**Published:** 2022-04-08

**Authors:** Liuxi Wu, Hua Shu, Wenqin Chen, Yingqian Gao, Ya Yuan, Xiao Li, Wenjuan Lu, Xinhua Ye, Hongyan Deng

**Affiliations:** 1 Department of Ultrasound, The First Affiliated Hospital of Nanjing Medical University, Nanjing, 210029 China; 2 Department of Pathology, The First Affiliated Hospital of Nanjing Medical University, Nanjing, 210029 China

**Keywords:** Biomarkers, Diseases, Medical research, Oncology

## Abstract

Fine needle aspiration biopsy is a crucial method for preoperative diagnosis of thyroid nodules. However, thyroid nodules classified as Bethesda categories III–V cannot obtain definite cytological results. Our aim was to study the diagnostic value of thyroid imaging reporting and data system combined with BRAF^V600E^ mutation analysis in Bethesda categories III–V thyroid nodules, so as to provide more precise direction for the follow-up treatments. A total of 174 Bethesda categories III–V thyroid nodules performed TIRADS and BRAF^V600E^ mutation analysis were included in the study. We retrospectively analyzed the ultrasound features as well as the results of BRAF^V600E^ mutation of the 174 thyroid nodules. In the multiple regression analysis models, ultrasound features including lobulated or irregular margin, punctate echogenic foci, and shape with taller-than-wide were statistically significant in malignant nodules (*p* < 0.05). The area under the curve of the combination of TIRADS and BRAF^V600E^ increased to 0.925, which were much higher than TIRADS (0.861) and BRAF^V600E^ (0.804) separately. Combined diagnosis was of the greatest value to identify Bethesda III–V thyroid nodules definitely, especially with higher sensitivity (93%) and accuracy (90%).

## Introduction

Fine needle aspiration (FNA) biopsy of the thyroid has been adopted as a fundamental procedure for diagnosing suspicious nodules indicated by Kwak’s Thyroid Imaging Reporting and Data System (TIRADS)^1^. Bethesda System^2^ recommended by the American thyroid association (ATA)^3^ to report the results of FNA. The Bethesda System showed a relatively high accuracy in reporting FNA cytology, with 89% of samples being satisfactory for interpretation, 74% reported as definitively benign and 5% as definitively malignant^4^. However, approximately 20–30% of the cytology samples still belong to indeterminate diagnostic results^5^, that is, Bethesda categories III–V (III: unclear cellular atypical lesion or unclear follicular lesion AUS/FLUS; IV: follicular lesion or suspicious follicular tumor SFN/FN; V: suspicious malignant tumor SM), with a certain degree of malignancy. Then, in order to obtain a definitive diagnosis, surgery is selected by a number of patients with nodules in these cytological categories^6^. Due to the aggressively biopsying small nodules and performing extensive surgeries, the detection rate of thyroid cancer has increased dramatically in recent decades^7^. However, the overall mortality rate of thyroid cancer is not significant^7^, and thyroid cancer remains one of the least deadly human cancers. With the increasing cost of surgery, which proves 4.7–6.5 times more expensive than active surveillance^8^, the excessive medical treatments in thyroid nodules are brought into focus. Therefore, it is imperative to diagnose malignant lesions among indeterminate thyroid nodules precisely to avoid unnecessary operations and achieve a good cost effectiveness.

To fulfill this goal, molecular marker profiling is performed as an auxiliary diagnosis of thyroid nodules. BRAF is a member of the raf family of serine/threonine protein kinases, which has been shown to be mutated in 7% of all cancers, promoting to the activation of downstream transcription factors, as well as leading to cell differentiation, proliferation, growth, and apoptosis^9^. Additionally, it is suggested that BRAF^V600E^ mutation is associated with poor prognosis of thyroid cancer^10^. The mutant BRAF^V600E^ gene has been reported in 83% thyroid cancers, approximately 60–70% differentiated thyroid carcinoma(DTC), 19–33% poorly differentiated thyroid carcinoma(PDTC) and 19–45% anaplastic thyroid carcinoma(ATC) thyroid carcinomas^11–14^. In a word, the utility of BRAF^V600E^ analysis for thyroid nodules enhances the diagnostic value of FNA cytology. However, in the diagnosis of indeterminate nodules, a previous study found that BRAF^V600E^ mutation seemed to play a limited role owing to its low sensitivity^15^.

To date, the value of TIRADS combined with BRAF^V600E^ mutation in distinguishing malignant from benign lesions in indeterminate thyroid nodules remains controversial. Wu et al.^6^ have demonstrated that BRAF^V600E^ mutation analysis and TIRADS classification were reliable diagnostic tools in Bethesda categories I(undiagnosed or unsatisfactory specimen ND/UNS), III, and V thyroid nodules in a Chinese population. Conversely, Bethesda category IV thyroid nodule was rarely associated with BRAF^V600E^ mutation^16^. Thus, this study aimed to evaluate the diagnostic values of TIRADS and BRAF^V600E^ mutation analysis in Bethesda categories III–V thyroid nodules, so as to provide clinical decision-making information for patients with thyroid nodules who failed to obtain a definite diagnosis after FNA.

## Results

### Clinical and ultrasonic features

A total of 172 patients including 42 males and 130 females with 174 indeterminate thyroid nodules (Bethesda categories III–V) were included in our research. The clinical and ultrasonic features are displayed in Table 1.

**Table 1:** Clinical and ultrasonic features of the thyroid nodules.

Variables, n (%)	Benign (n = 74)	Malignant (n = 100)	p*	Odds Ratio (95% CI)	*p*^§^
**Shape**					
Wider-than-tall	68 (92)	61 (61)	< 0.001	1 (reference)	0.002
Taller-than-wide	6 (8)	39 (39)		6.117 (1.936,19.322)	
**Echogenicity**					
Isoechoic/Hyperechoic	4 (5)	10 (10)	0.008	1 (reference)	0.1
Hypoechoic	53 (72)	83 (83)		3.546 (0.548–22.943)	84
Very hypoechoic	17 (23)	7 (7)		1.816 (0.504–6.541)	0.361
**Margin**					
Smooth	46 (62)	21 (21)	< 0.001	1 (reference)	
Lobulated or irregular	9 (12)	38 (38)		4.178 (1.511,11.552)	0.006
Ill-defined	19 (26)	41 (41)		2.318 (0.942,5.706)	0.067
**Echogenic foci**					
None	23 (31)	15 (15)	0.016	1 (reference)	
Punctate echogenic foci	38 (51)	74 (74)		3.05 (1.096–8.490)	0.033
Macrocalcifications	10 (14)	7 (7)		1.736 (0.406–7.429)	0.457
Peripheral calcifications	3 (4)	4 (4)		4.354 (0.557–34.044)	0.161
**Acoustis halo**					
None	62 (84)	96 (96)	0.004	1 (reference)	
Complete	10 (14)	1 (1)		0.294 (0.03–2.842)	0.290
Interrupted	2 (3)	3 (3)		3.648 (0.403–33.058)	0.250
**Blood flow**					
None	17 (23)	41 (41)	0.029	1 (reference)	
Hypervascular	30 (41)	26 (26)		0.768 (0.283–2.082)	0.603
Mild/moderate	27 (36)	33 (33)		0.756 (0.305–1.871)	0.545
**Posterior echo**					
None	72 (97)	90 (90)	0.060		
Attenuation	2 (3)	10 (10)			
**Composition**					
Solid	69 (93)	97 (97)	0.287		
Mixed cystic and solid	5 (7)	3 (3)			
Age, years (mean ± SD)	47 ± 14	44 ± 13	0.146		
**Gender**					
Male	14 (19)	29 (29)	0.127		
Female	60 (81)	71 (71)			
Diameter, milimeters (mean ± SD)	18 ± 13	12 ± 7	< 0.001		

*By chi-square test.

^§^By multiple logistic regression analysis.

The mean size of malignant nodules were 12 ± 7 mm, which displayed significantly smaller maximal diameters than benign ones (*p* < 0.001). In univariate analysis, shape, echogenicity, margin, echogenic foci, acoustic halo, and blood flow proved statistical significance between benign and malignant nodules (*p* < 0.05). In multiple regression analysis, shape with taller-than-wide, punctate echogenic foci, lobulated or irregular margin were ultrasonography features showing significant statistically (*p* < 0.05).

### The diagnostic efficacy of TIRADS, BRAF^V600E^ mutation analysis and combined diagnosis in Bethesda categories III–V thyroid nodules.

**Figure 1:**
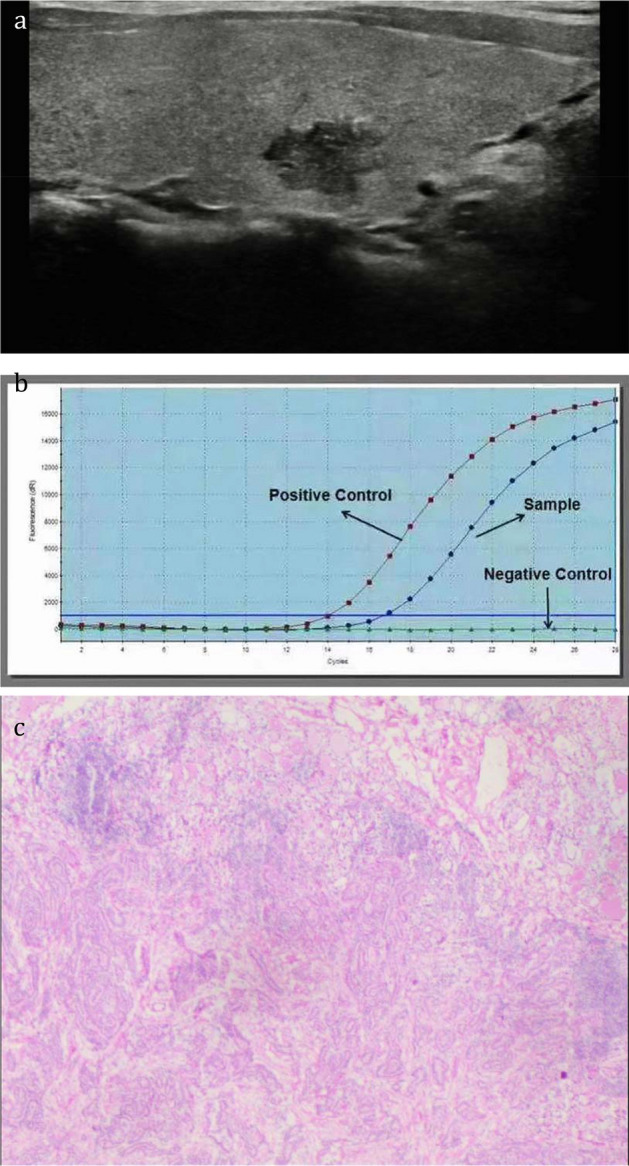
A thyroid nodule, assigned to Bethesda category III, was considered malignant diagnosed by the combination of TIRADS and BRAF^V600E^ analysis. The nodule proved to be a malignant tumour confirmed by postoperative pathology. (**a**), the 2D sonogram classified as TIRADS 4b, (**b**), mutant BRAF^V600E^ gene, (**c**), papillary thyroid carcinoma.

**Figure 2:**
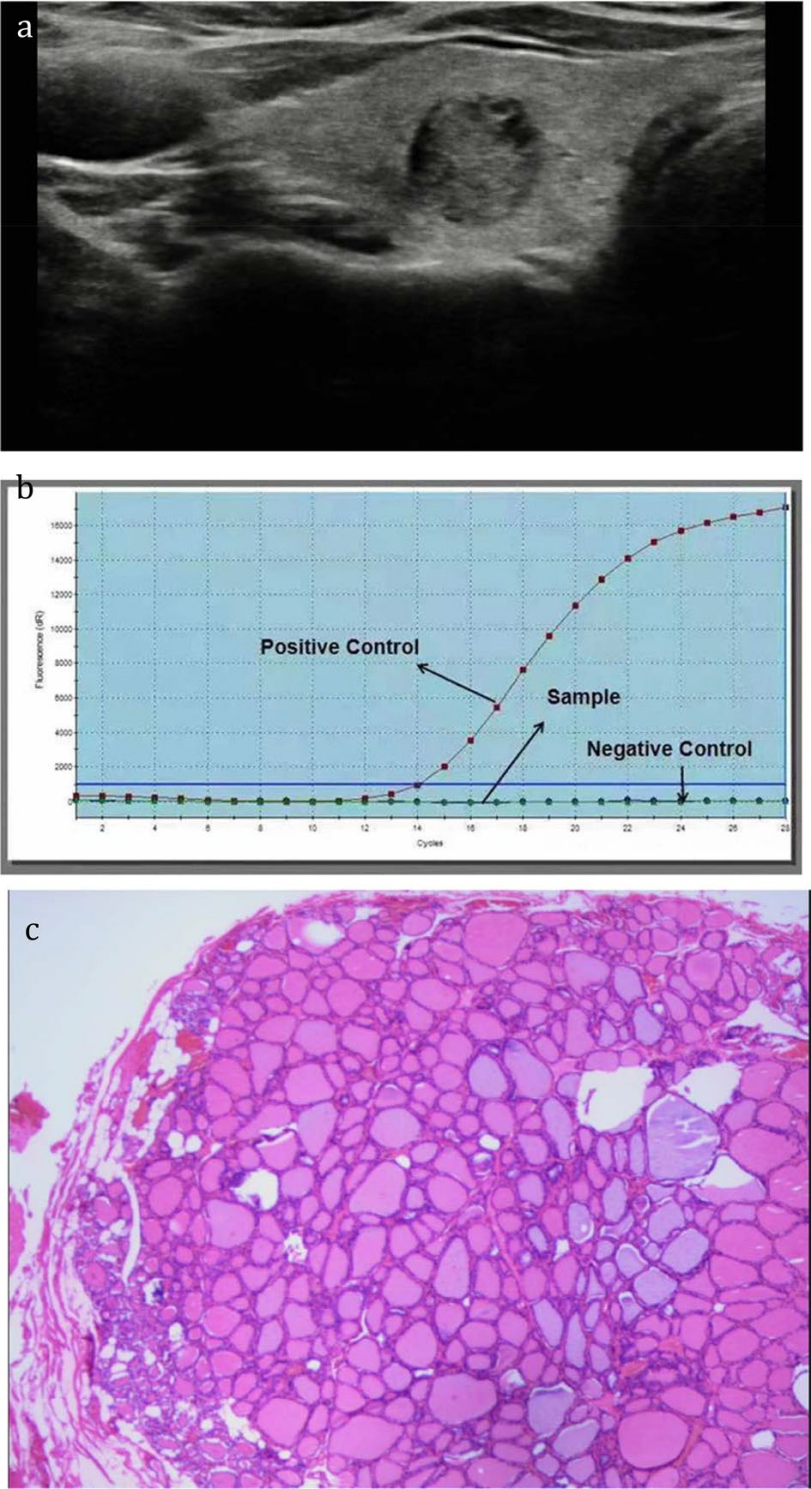
A thyroid nodule, assigned to Bethesda category III, was considered benign diagnosed by the combination of TIRADS and BRAF^V600E^ analysis. The nodule proved to be a benign one confirmed by postoperative pathology. (**a**), the 2D sonogram classified as TIRADS 4a, (**b**), wild-type BRAF^V600E^ gene, (**c**), nodular goiter confirmed by postoperative pathology.

**Figure 3:**
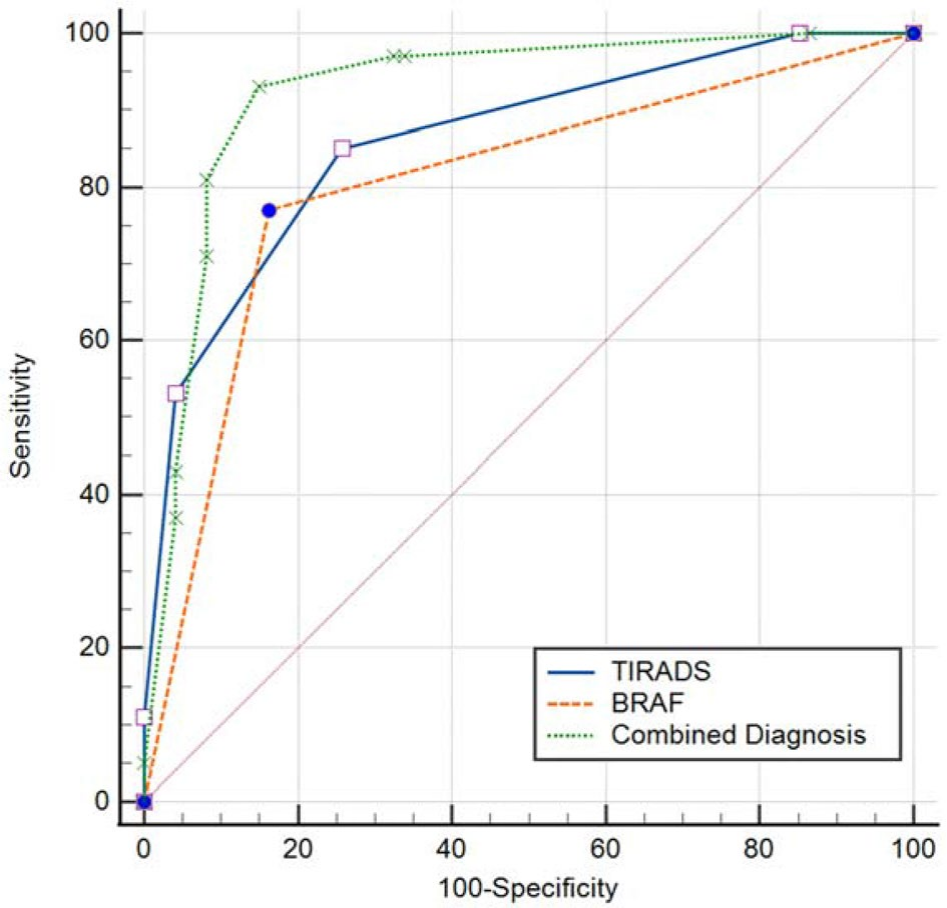
The AUC of combined diagnosis, TIRADS, BRAF^V600E^: 0.925, 0.861, and 0.804(*p* < 0.05).

**Figure 4:**
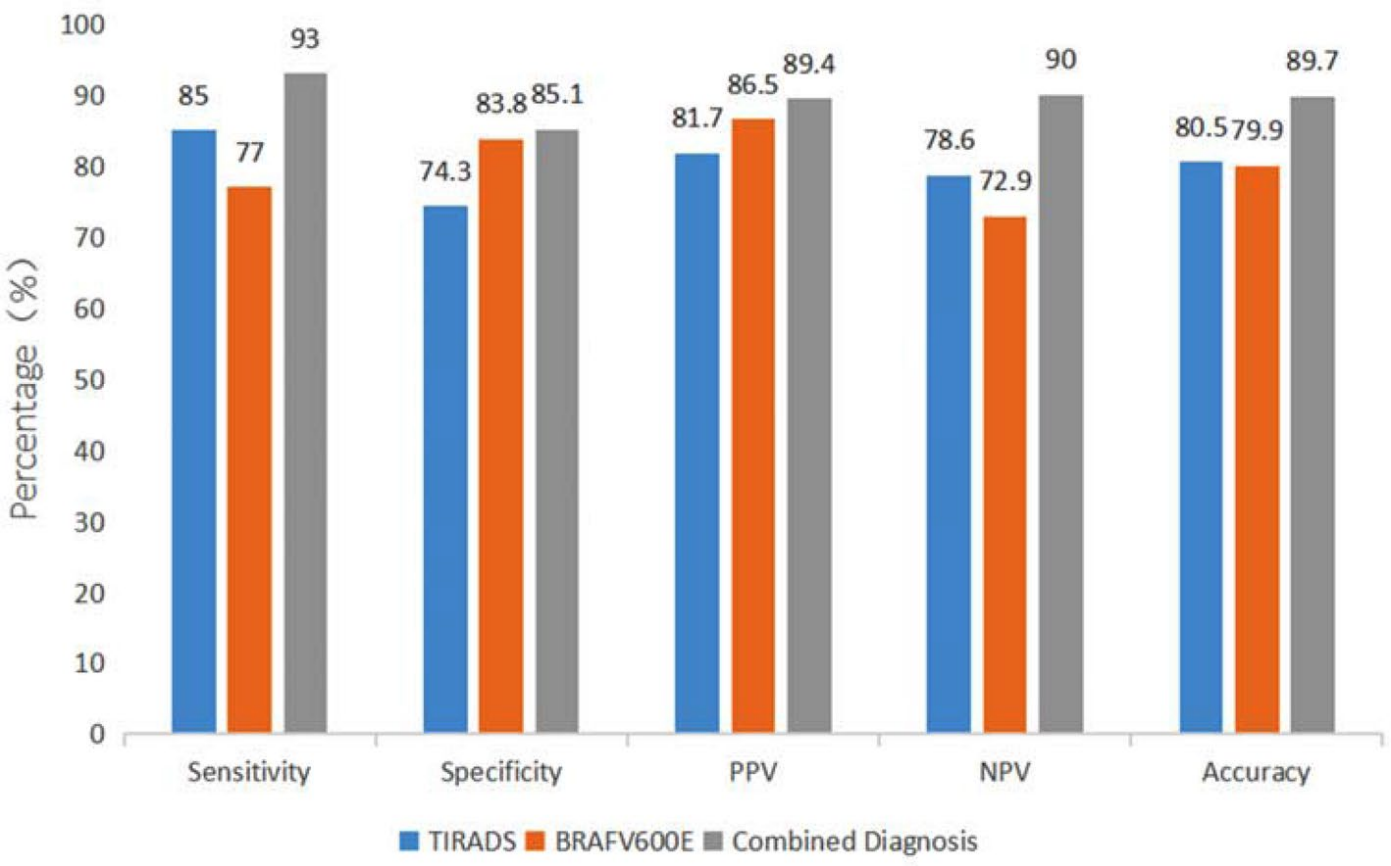
Comparison of diagnostic performance of TIRADS, BRAF^V600E^, and combination of the two.

**Figure 5:**
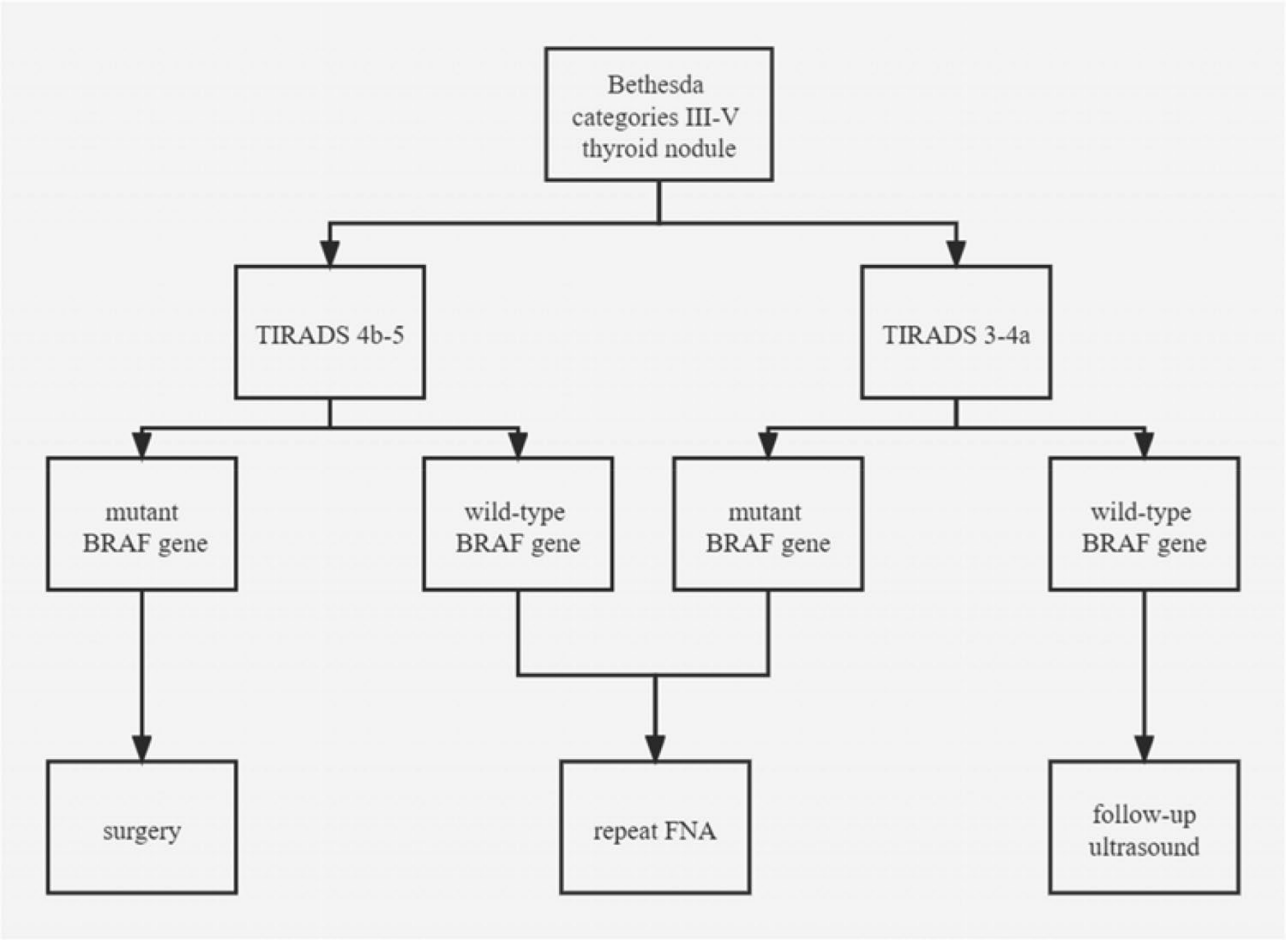
Managements of Bethesda categories III–V thyroid nodules.

The specific information of thyroid nodules from different diagnostic methods is presented in Table 2. Cases diagnosed by TIRADS and BRAF^V600E^ are displayed in Figs. 1 and 2. In Bethesda categories III–V nodules, the ROC curve showed that the best cut-off for TIRADS classification was 4b. Therefore, cases with TIRADS classification 4b-5 would be regarded as cancers. The area under the curve (AUC) of TIRADS classification was 0.861, with 85% sensitivity, 74.3% specificity, 81.7% positive predictive value (PPV), 78.6% negative predictive value (NPV) and 80.5% accuracy. A total of 77 nodules with BRAF^V600E^ mutation were considered as malignancy. The AUC of BRAF^V600E^ mutation test was 0.804, with 77% sensitivity, 83.8% specificity, 86.5% PPV, 72.9% NPV, and 79.9% accuracy. The AUC of the combination of the TIRADS and BRAF^V600E^ was 0.925, with 93% sensitivity, 85.1% specificity, 89.4% PPV, 90.0% NPV, and 89.7% accuracy, which were much higher than those of TIRADS and BRAF^V600E^ separately in predicting malignant thyroid nodules (*p* < 0.05)(Figs. 3, 4). On the basis of the combined diagnosis, managements of Bethesda categories III–V thyroid nodules is demonstrated in Fig. 5.

**Table 2:** The specific information of Bethesda categories III–V thyroid nodules adopting TIRADS, BRAF^V600E^ and combined diagnosis.

	Benign (n = 74)	Malignant (n = 100)
**TIRADS**		
3 (n = 11)	11	0
4a (n = 59)	44	15
4b (n = 48)	16	32
4c (n = 45)	3	42
5 (n = 11)	0	11
**BRAF**^**V600E**^		
Wild type (n = 85)	62	23
Mutant type (n = 89)	12	77
**Combined diagnosis**		
Benign (n = 70)	63	7
Malignant (n = 104)	11	93

## Discussion

It was reported that the histopathology malignancy rates was 98% for cytology malignant cases (Bethesda category VI) through FNA^17^. Cha et al. found the malignant rates in surgical cases are as follows for Bethesda category: III (50.6%), IV (52.3%), and V (90.7%)^18^, and meanwhile our study indicated that the malignant risks of III, IV(follicular adenoma confirmed by the postoperative pathology) and V were 34.6%, 0% and 80.2%, respectively, which were higher than the estimated malignant risks of the Bethesda system in III and V nodules^2^, indicating the conservative approaches of pathologists. These three categories of Bethesda system are full of uncertainty and are managed entirely differently by a number of centers. Therefore, appropriate diagnostic methods were required to distinguish malignant and benign lesions among these nodules. The present study discovered that the combination of TIRADS and BRAF^V600E^ mutation analysis achieved a better diagnostic efficiency in differentiating Bethesda categories III–V thyroid nodules.

A study indicated that the incidence rate of thyroid cancer has increased significantly among the population aged 15–39, especially in women^19^. In our research, malignant thyroid nodules has a higher incidence in women than men and the average age of patients subjected to thyroid cancer was 44, which is consistent with Lim’s finding^20^. Thereby, females with questionable thyroid nodule are supposed to be treated more carefully.

Ultrasound is the primary and preferred examination method for risk stratification of thyroid nodules, which could guide the follow-up measures. We figured out that the maximum diameter of a benign nodule were larger than a malignant one (*p* < 0.001), cohering with the Kwak’s study^21^. In our multiple logistic regression analysis, shape with taller than wide, lobulated or irregular margin, and punctate echogenic foci were independent risk factors (*p* < 0.05), consistent with previous studies^22–24^. It was certain that the above-mentioned ultrasound signs could play a vital role in diagnosing indeterminate thyroid nodules. In current study, the ROC curve showed that the best cut-off for TIRADS classification was 4b, consistent with findings perceived by Zhang et al.^25,26^. Nevertheless, our research suggested a slightly higher sensitivity of TIRADS compared to the Zhang’s discovery which included Bethesda categories I–VI (85% versus 73.1%) and a higher PPV compared to a finding by Singaporewalla et al.^27^(81.7% versus 60%). According to the guidelines, the risks of malignancy of nodules classified as TIRADS 3, 4a, 4b, 4c, and 5 were < 2%, 2–10%, 10–50%, 50–95%, and ≥ 95%, respectively^1^, consistent with our findings in part. The malignant rates of TIRADS 4a, and 4b were 25.4% (15/59), and 66.7% (32/48) in our study, which were higher than the guideline^1^. This was probably on account of the cautious assessments of sonographers and differences in populations. At the same time, our data demonstrated that 95.4% (166/174) thyroid nodules belonged to solid nodules, which had no statistically significance in the differential diagnosis of benign and malignant nodules. In practice, a solid thyroid nodule without other suspicious ultrasonic characteristics was inclined to a benign one classified as TIRADS 3 in our institution. Even though there were limitations in TIRADS, it still provided a standardized risk stratification for thyroid nodules to guide follow-up managements for clinicians.

The prevalence of BRAF mutation in thyroid cancer was 77% (77/100), in our series which was comparable to another report from Kim et al.^28^. A correlation between the BRAF^V600E^ mutation and aggressive disease features, including lymph node metastases, invasion, and recurrence, has been reported by Martina et al.^29^. BRAF analysis boosted the accuracy of cytology and possessed a particular value for indeterminate nodules in the Chinese population^30^. In our study, the malignant rate of nodules with BRAF^V600E^ mutation was 86.5% (77/89) in Bethesda categories III–V, consistent with findings by Zhu et al.^31,32^. Seo et al.^33^ found that BRAF mutation analysis showed additional diagnostic value in thyroid nodules with Bethesda category V alone even when the nodules do not show suspicious ultrasonic features. A meta-analysis of 32 studies found that the overall specificity for BRAF^V600E^ in the diagnosis of thyroid cancers was 100% in indeterminate nodules^15^, conforming to a finding by Han et al.^34^. In general, BRAF^V600E^ gene had a significant difference in Bethesda categories III–V thyroid nodules. If the indeterminate thyroid nodule with a mutant BRAF^V600E^ gene, repeat FNA might be considered to supplement malignancy risk assessment^3^.

The diagnostic sensitivity, accuracy, and AUC of the combined diagnosis were much higher than those of TIRADS or BRAF^V600E^ separately, in accordance with Wu’s finding^6^, improving the ability to diagnose malignant nodules and reduce false negatives. The malignant rate of thyroid nodules with BRAF^V600E^ mutation and TIRADS classification 4b-5 was up to 89.4% (93/104) in our study, similar to the malignant rate of Bethesda category VI thyroid nodules^35^. Accordingly, TIRADS combined with BRAF^V600E^ mutation analysis reached a valuable diagnostic efficacy for Bethesda III–V thyroid nodules.

There were several limitations in the our study. Firstly, this study was a retrospective study. Patients with nodules of Bethesda categories III–IV mostly chose follow-up surveillance, and meanwhile, a part of patients with Bethesda categories V nodules turned to surgical treatment because of factors such as large nodules, mental tension, and mutant BRAF^V600E^ gene, resulting in a certain degree of selection bias. Secondly, the single center with a small sample, which is still needed to be further verified by expanding the volume of sample and cooperating with other institutions. Lastly, we did not explore the predictive value of TIRADS and BRAF^V600E^ in the staging and prognostic of thyroid carcinomas, which has been confirmed in another study^29^.

In summary, the combination of TIRADS and BRAF^V600E^ had the highest diagnostic efficacy in Bethesda categories III–V thyroid nodules. For Bethesda categories III–V thyroid nodules with BRAF^V600E^ mutation and TIRADS classification 4b-5, surgery should be recommended. Otherwise, regular follow-up ultrasound or repeat FNA were deemed to be appropriate.

## Methods

### Patients

This study enrolled a total of 172 patients aged from 17 to 76 with 174 indeterminate thyroid nodules who underwent FNA, two dimension (2D) ultrasonography and BRAF^V600E^ mutation analysis in the First Affiliated Hospital of Nanjing Medical University from February 2018 to November 2021. Inclusion criteria were: (1) complete clinical and ultrasound data; (2) nodules in Bethesda categories III–V. Exclusion criteria were: (1) failure to complete any of 2D ultrasonography, FNA and BRAF^V600E^ mutation analysis; (2) Bethesda categories III–V nodules with increased size (≥ 20%) in any dimension by follow-up ultrasound with no surgical histopathology results.

This retrospective study was approved by the Institutional Ethical Committee in the First Affiliated Hospital of Nanjing Medical University and written informed consent was obtained from all the patients before performing FNA and BRAF^V600E^ analysis. All methods were conducted in accordance with the relevant guidelines and regulations.

### Ultrasonography and TIRADS

Philips Epiq 5 (L12-5, 5–12 MHz) and Super Sonic Imagine Aixplorer-1 (SL15-4, 4–15 MHz) were used for routine 2D ultrasound examination. The size, shape, composition, echo, blood flow and other parameters of nodules were collected. According to TIRADS proposed by Kwak et al.^1^, malignant ultrasound features include solid components, hypoechogenicity or marked hypoechogenicity, lobulated or irregular margins, punctate echogenic foci, and taller-than-wide shape. Based on the presence of ultrasonic risk features, each thyroid nodule was classified into 1–5 grades: TIRADS 3 (no suspicious characteristics), TIRADS 4a (1 suspicious characteristic), TIRADS 4b (2 suspicious characteristics), TIRADS 4c (3 or 4 suspicious characteristics), and TIRADS 5 (5 suspicious characteristics). The classification of thyroid nodules was performed by two ultrasound physicians with more than 5 year experience using TIRADS. Disagreement was discussed with the help of another more advanced physician until consensus was reached.

### FNA and Bethesda

GE Logiq-E9 (ML6-15, 12 MHz) color Doppler ultrasound diagnostic instrument was used for ultrasound-guided fine needle biopsy. FNA of the thyroid nodules was performed with 25-gauge needles. The samples of FNA were examined for cytology in the pathology department and the reports of cytology was classified based on Bethesda thyroid reporting system (category I: undiagnosed or unsatisfactory specimen ND/UNS; II: benign lesion B; III: unclear cellular atypical lesion or unclear follicular lesion AUS/FLUS; IV: follicular lesion or suspicious follicular tumor SFN/FN; V: suspicious malignant tumor SM; VI: malignant tumor/M).

### BRAF ^V600E^ mutation annalysis

Polymerase Chain Reaction (PCR) was used to detect BRAF^V600E^. PCR primers were designed according to BRAF sequence to obtain cycle threshold (CT). Those with CT value less than 28 were positive and those with CT value greater than or equal to 28 were negative.

### Statistics

The statistical analysis was done using the Statistical Package for the Social Sciences (SPSS) software version 26.0. Categorical variables were reported as percentages and analysed with Pearson’s χ2 or Fisher’s exact test when applicable. Continuous variables were expressed as mean with standard deviation when normally distributed and subjected to the two-sample *t*-test. Multiple logistic regression analysis was performed to determine variables that correlated with a malignant nodule. Receiver operating characteristic (ROC) curves of TIRADS, BRAF^V600E^ and combined predictor were drawn by MedCalc 20 software. The area under the curve (AUC) was compared to evaluate the diagnostic efficacy of TIRADS, BRAF^V600E^ and both of them in Bethesda categories III–V thyroid nodules. A *p* value of < 0.05 was considered statistically significant.

## Data Availability

The datasets generated and analysed during the current study are available from the corresponding author on reasonable request.
